# Combined defects in oxidative phosphorylation and fatty acid β-oxidation in mitochondrial disease

**DOI:** 10.1042/BSR20150295

**Published:** 2016-03-16

**Authors:** Abena Nsiah-Sefaa, Matthew McKenzie

**Affiliations:** * Centre for Genetic Diseases, Hudson Institute of Medical Research, Clayton, Victoria 3168, Australia; † The Department of Molecular and Translational Science, Monash University, Clayton, Victoria 3168, Australia

**Keywords:** disease, mitochondria, protein complex assembly, protein interactions, supercomplex

## Abstract

Mitochondria provide the main source of energy to eukaryotic cells, oxidizing fats and sugars to generate ATP. Mitochondrial fatty acid β-oxidation (FAO) and oxidative phosphorylation (OXPHOS) are two metabolic pathways which are central to this process. Defects in these pathways can result in diseases of the brain, skeletal muscle, heart and liver, affecting approximately 1 in 5000 live births. There are no effective therapies for these disorders, with quality of life severely reduced for most patients. The pathology underlying many aspects of these diseases is not well understood; for example, it is not clear why some patients with primary FAO deficiencies exhibit secondary OXPHOS defects. However, recent findings suggest that physical interactions exist between FAO and OXPHOS proteins, and that these interactions are critical for both FAO and OXPHOS function. Here, we review our current understanding of the interactions between FAO and OXPHOS proteins and how defects in these two metabolic pathways contribute to mitochondrial disease pathogenesis.

## MITOCHONDRIAL METABOLISM

Mitochondria occupy almost all human cell types and are involved in essential metabolic and cellular processes, including calcium and iron homoeostasis, apoptosis, innate immunity and haeme biosynthesis [1]. However, the primary function of mitochondria is the production of energy in the form of ATP [2–4]. ATP is the body's energy currency, playing vital roles in cell differentiation, growth and reproduction, thermogenesis and powering the contraction of muscles for movement [1]. In humans, ATP is produced by two different processes; through the breakdown of glucose or other sugars in the absence of oxygen in the cytoplasm (glycolysis), or by the metabolism of fats, sugars and proteins in the mitochondria in the presence of oxygen. Although both processes produce ATP, oxidative metabolism accounts for 95% of ATP produced and yields 20 times the amount of ATP as its anaerobic counterpart.

**Figure 1:**
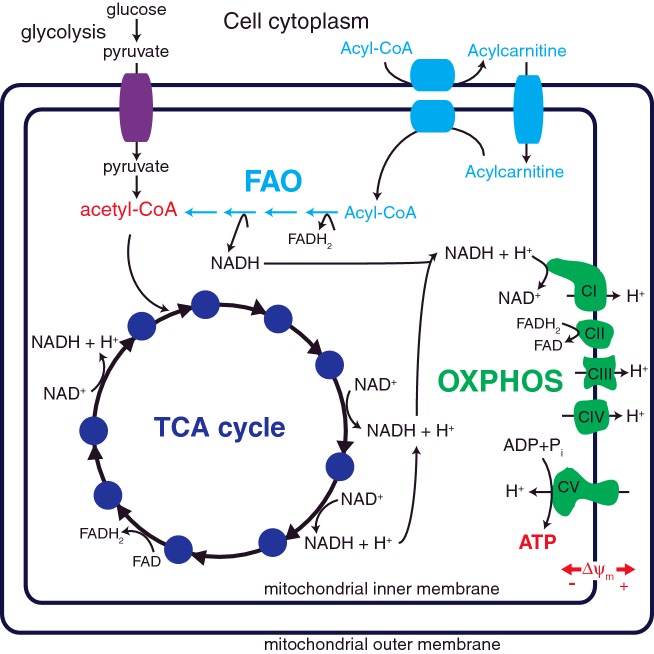
Mitochondrial metabolism Glucose breakdown through glycolysis and the TCA cycle (dark blue) generates reduced NADH and FADH_2_. Fatty acid β-oxidation (FAO, light blue) of fatty acyl-CoA esters is performed in four enzymatic reactions that also generates NADH and FADH_2_, as well as acetyl-CoA. Electrons derived from NADH and FADH_2_ are utilized by the five OXPHOS complexes (green) to generate ATP. Complex I (CI, NADH: ubiquinone oxidoreductase), complex III (CIII, ubiquinol: ferricytochrome *c* oxidoreductase) and complex IV (CIV, cytochrome *c* oxidase) pump electrons out of the mitochondrial matrix to generate a membrane potential (Δ*ψ*_m_) that drives the synthesis of ATP by complex V (CV, F_o_F_1_-ATP synthetase). CII, complex II (succinate: ubiquinone oxidoreductase).

Mitochondria utilize three main enzymatic pathways to generate ATP; the tricarboxylic acid (TCA) cycle, oxidative phosphorylation (OXPHOS) and fatty acid β-oxidation (FAO). The TCA cycle oxidizes acetyl-CoA, derived from sugars, fats and amino acids, to generate NADH and flavin adenine dinucleotide (FADH_2_), which can be used by the OXPHOS system to generate ATP (Figure 1).

## OXIDATIVE PHOSPHORYLATION (OXPHOS)

OXPHOS involves a series of oxidation–reduction reactions which results in the phosphorylation of ADP to produce ATP. This process is performed by five protein complexes which reside in the inner mitochondrial membrane: complex I (CI), NADH: ubiquinone oxidoreductase, EC 1.6.5.3; complex II (CII), succinate: ubiquinone oxidoreductase, EC 1.3.5.1; complex III (CIII) ubiquinol: ferricytochrome *c* oxidoreductase, EC 1.10.2.2; complex IV (CIV) cytochrome *c* oxidase, EC 1.9.3.1 and complex V (CV), F_o_F_1_-ATP synthetase, EC; 3.6.3.14.

Complex I (CI) accepts electrons from NADH, whereas CII accepts electrons from FADH_2_, both of which are derived from the TCA cycle and FAO. CI and CII then reduce ubiquinone, the substrate of CIII. CIII then transfers electrons from reduced ubiquinone to cytochrome *c*. Next, CIV passes the electrons from cytochrome *c* to O_2_, reducing it to form H_2_O. As the electrons are transferred between the OXPHOS complexes, protons are pumped across the inner mitochondrial membrane by CI, CIII and CIV to create an electrochemical potential (∆*ψ*_m_). ∆*ψ*_m_ is used by CV to drive the phosphorylation of ADP to produce ATP (Figure 1).

## OXPHOS SUPERCOMPLEXES

Since the identification of the five OXPHOS complexes, their orientation *in situ* has been debated. Two main theories have been postulated; the fluid model and the solid-state model [5]. The fluid model suggests that the OXPHOS complexes localize individually and diffuse laterally in the mitochondrial inner membrane. In this model, electron transfer is dependent on random collisions between CI, CII, CIII and CIV [6]. Conversely, the solid-state theory proposes that the constituents of OXPHOS combine to form stable structures, termed ‘supercomplexes’, that contain two or more of the OXPHOS complexes.

Initial findings identified possible physical interactions between CI and CIII [7,8], as well as CII and CIII [9]. Further research resulted in the isolation of a supercomplex containing CIII/CIV_2_ in several strains of bacteria [10–12] and CI/CIII_2_, CIII_2_, CI_2_/CIV and CI/CIII_2_/CIV with varying stoichiometry in potato mitochondria [13,14]. In yeast, a CV dimer (CV_2_) [15] and CIII_2_/CIV_1–2_ supercomplex have also been detected [16].

In mammalian mitochondria, CI/CIII_2_ and CI/CIII_2_/CIV_1-3_ supercomplexes have been identified. In particular, the CI/CIII_2_/CIV_1–3_ supercomplex has been described as the ‘respirasome’, representing a single, functional respiratory unit [16]. Although the existence of OXPHOS supercomplexes within the inner mitochondrial membrane is now widely accepted, the function of these structures is still debated. A large body of research has revealed that the supercomplexes are integral for OXPHOS complex stability. Experimental evidence showed that a mutation in *MT-CYB*, which encodes the cytochrome *b* subunit of CIII, results in the disruption of CIII assembly in both mice and humans. In addition, destabilization of CI was also observed. This suggests that the presence of CIII is essential for CI assembly and/or stability via their interaction in a supercomplex [17].

Similarly, knockout of one of the CIV assembly factor genes, *COX10*, results in the loss of CIV activity and steady-state levels, with an associated reduction in mitochondrial respiratory capacity. Perturbed assembly of CI was also observed in *COX10* knockout mouse mitochondria, suggesting that stable CIV is required to maintain CI stability [18]. Overall, these findings highlight the interdependence of the OXPHOS complexes for their stability, via their association in the OXPHOS supercomplex.

In addition, the phospholipid cardiolipin is required for OXPHOS supercomplex assembly and stability [19]. The majority of cardiolipin is found in the inner mitochondrial membrane where it is essential for mitochondrial function. Nuclear magnetic resonance imaging of bovine heart mitochondria has identified cardiolipin attached to CV [20]. It has also been shown that cardiolipin is required to maintain CI, CIII and CIV structure and function [21].

In humans, mutations to *TAZ*, which encodes the cardiolipin acyltransferase Tafazzin, result in Barth syndrome, a disease characterized by dilated cardiomyopathy, skeletal myopathy and neutropenia [22]. Barth syndrome patients exhibit increased accumulation of monolysocardiolipin precursors and reduced mature tetralinoleoylcardiolipin production, with associated CIII and CIV deficiencies [23]. It has also been shown that Tafazzin defects result in destabilized OXPHOS supercomplexes, which in turn results in reduced steady-state levels of CI. These findings suggest that cardiolipin is essential for OXPHOS supercomplex stability, and that loss of supercomplex stability contributes to Barth syndrome pathogenesis [19].

Apart from stabilizing the OXPHOS complexes, the supercomplex structure may also play a role in substrate channelling. Flux experiments have shown that the formation of the respirasome allows for substrate channelling by decreasing the distance in which electrons travel between mobile electron carriers and the OXPHOS complexes. This finding is supported by in-gel enzymatic assays that demonstrate respirasome catalytic activity in a range of eukaryotes [16,24,25]. In addition, the formation of the respirasome has also been proposed to limit oxidative stress [26]. By forcing closer interactions between CI and CIII, the leakage of electrons to form superoxide is less likely. Indeed, oxidative stress is a common attribute in diseases where supercomplex assembly is disturbed (reviewed in [27,28]).

However, recent findings suggest that the main function of supercomplexes is not to channel substrates and stabilize the OXPHOS complexes, but may instead be a protein packaging and space saving phenomenon. Recent flux control analyses have discounted electron channelling in supercomplexes [29], while electron microscopy of the supercomplex structure has revealed that the distances between CIII and CIV may be too large for efficient substrate channelling [30]. In addition, it has been proposed that the interdependence of complex stability is most likely due to downstream effects of increased oxidative stress and not due to their presence in the same supercomplex [29].

In summary, although the existence of OXPHOS supercomplexes is now largely established, there is still debate regarding their functional significance. Irrespective of their purpose, the OXPHOS supercomplexes play an important role in mitochondrial respiration and their disruption contributes to mitochondrial disease pathogenesis, possibly in ways we are yet to fully understand.

## MITOCHONDRIAL FATTY ACID β-OXIDATION (FAO)

Fatty acids are vital constituents of enzymes, hormones and cell membranes. In addition, they are a major source of energy. Fatty acids are metabolized in mitochondria by FAO, a critical pathway of energy production in a variety of cell types, including the heart [31,32]. In fact, under normal physiological conditions, FAO provides the majority of ATP (60–70%) required for proper heart contraction [33]. FAO yields a high amount of energy for the cell. For example, the complete oxidation of a 16-carbon palmitic acid will yield a total of 112 molecules of ATP.

FAO is crucial for homoeostatic regulation, specifically in times of fasting or endurance exercise that requires high energy resources. During this high energy demand, fat stores are broken down for metabolism by tissues in need [34]. In particular, the liver metabolizes fatty acids to produce ketone bodies for consumption by the brain when glucose is unavailable [35,36].

At least 20 separate transport proteins and enzymes are required for activation and breakdown of fatty acids via FAO (Table 2) [37]. Fatty acids are transported through the blood as non-esterified fatty acids bound to lipoproteins or serum albumin. Upon reaching their target cell, short and medium chain fatty acids (C_4_–C_12_) traverse the cell membrane by passive diffusion. However, saturated and unsaturated long chain fatty acids cross the cell membrane by sodium dependent fatty acid transporters [38]. These include fatty acid transport proteins (FATPs), plasma membrane fatty acid binding proteins and the fatty acid translocase protein CD36.

**Figure 2:**
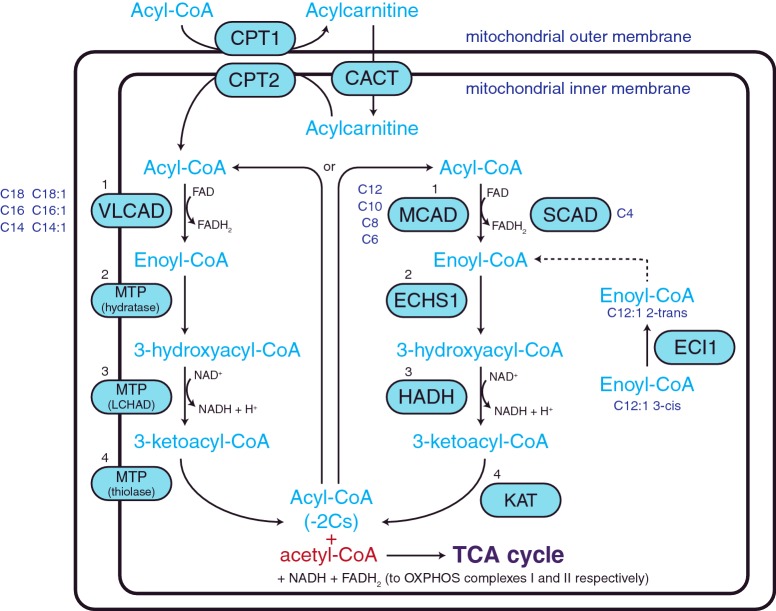
Mitochondrial fatty acid β-oxidation (FAO) spiral Fatty acyl-CoA esters are converted to fatty acylcarnitines by CPT1 for transport into the mitochondria by CACT. Acylcarnitines are subsequently converted back to fatty acyl-CoA esters once inside the mitochondria by CPT2 for metabolism by the fatty acid β-oxidation (FAO) spiral. FAO consists of four reactions (numbered 1–4 in black) which are performed by enzymes that are fatty acid chain length specific (chain lengths shown in dark blue). (1) Dehydrogenation of the fatty acyl-CoA by very long chain (VLCAD), medium chain (MCAD) or short chain (SCAD) acyl-CoA dehydrogenases to create enoyl-CoA, (2) hydration by the enoyl-CoA hydratase activity of the MTP or ECHS1 to add water to enoyl-CoA to form 3-hydroxyacyl-CoA, (3) a second dehydrogenation by MTP or HADH to generate 3-ketoacyl-CoA and (4) thiolysis by the thiolase activity of the MTP or KAT to produce a shortened fatty acyl-CoA and acetyl-CoA. Oxidation of unsaturated fatty acids requires the action of ECI1.

Once inside the cell, acyl-CoA synthetases activate the fatty acid by converting it from its non-esterified form to a fatty acyl-CoA ester. These esters can form the preliminary substrates for cholesterol, phospholipid and triacylglycerol synthesis, or enter the mitochondria via the carnitine shuttle system for FAO (Figure 2). The carnitine transport of fatty acyl-CoAs involves three steps. Firstly, the fatty acyl-CoA is bound to carnitine by carnitine O-palmitoyltransferase 1 (CPT1) to form a fatty acylcarnitine. Fatty acylcarnitines are then transported across the mitochondrial inner membrane by the carnitine acylcarnitine translocase (CACT). Once inside the mitochondrial matrix, carnitine O-palmitoyltransferase 2 (CPT2) converts the fatty acylcarnitine back to a fatty acyl-CoA ester [4].

Metabolism of fatty acyl-CoAs by FAO requires four enzymatic reactions; dehydrogenation, hydration, a second dehydrogenation and thiolysis. The end products of these reactions are one acetyl-CoA molecule, two electrons (which enter OXPHOS) and a fatty acyl-CoA ester that has been shortened by two carbon atoms. As such, the FAO pathway is often referred to as a spiral pathway, as the resulting shortened fatty acyl-CoA ester returns to the beginning of the pathway and is re-oxidized until only two acetyl-CoA molecules remain (Figure 2).

In the first step of FAO, fatty acyl-CoAs undergo dehydrogenation, removing two hydrogen atoms to create enoyl-CoA. Dehydrogenation is performed by a family of acyl-CoA dehydrogenases with particular fatty acid chain length specificity, ranging from 4 to 24 carbons. This family of enzymes includes: very long chain acyl-CoA dehydrogenase (VLCAD, C_12_–C_24_), long chain acyl-CoA dehydrogenase (LCAD, C_14_–C_18_) (low expression in humans), medium chain acyl-CoA dehydrogenase (MCAD, C_6_–C_12_) and short chain acyl-CoA dehydrogenase (SCAD, C_4_–C_6_). Dehydrogenation is flavin adenine dinucleotide (FAD) dependent, with FAD reduced to FADH_2_. The liberated electrons are passed from FADH_2_ to the electron transfer flavoprotein (ETF), a heterodimer consisting of an alpha (ETFA) and beta (ETFB) subunit. The electrons are then transferred to ubiquinone by the ETF: ubiquinone oxidoreductase (ETF-QO). Finally, ubiquinone enters the OXPHOS pathway by being oxidized by CIII.

For long chain fatty acids (C_14_–C_18_), the remaining three steps are catalysed by the mitochondrial trifunctional protein (MTP). Encoded by two genes, MTP is a hetero-octamer consisting of four α-subunits (encoded by *HADHA*) and four β-subunits (encoded by *HADHB*). The α-subunit comprises long chain enoyl-CoA hydratase (LCEH) and the NAD-dependent enzyme long chain 3-hydroxyacyl-CoA dehydrogenase (LCHAD), whereas long chain 3-ketoacyl-CoA thiolase (LKAT) is found in the β-subunit.

For medium (C_6_–C_12_) and short chain fatty acyl-CoAs (C_4_–C_6_), the last three steps of FAO are performed by different enzymes that are located in the mitochondrial matrix. These include: step 2, hydration by short chain enoyl-CoA hydratase 1 (ECHS1 or crotonase), step 3, dehydrogenation by hydroxyacyl-CoA dehydrogenase (HADH) and step 4, thiolysis by 3-ketoacyl-CoA thiolase (KAT) (Figure 2).

Fatty acids that have an uneven number of carbon atoms result in a three-carbon propanoyl-CoA at the last spiral, which is subsequently converted to succinyl-CoA in three steps. Propanoyl-CoA is carboxylated to (*S*)-methylmalonyl-CoA by propionyl-CoA carboxylase (PCC), a dodecameric enzyme comprised of six alpha (PCCA) and six beta (PCCB) subunits. (*S*)-Methylmalonyl-CoA is then isomerized by methylmalonyl-CoA epimerase (MCEE) to form (R)-methylmalonyl-CoA. The final step is performed by methylmalonyl-CoA mutase (MCM) and requires co-factor Vitamin B12 to produce succinyl-CoA, which then enters the TCA cycle.

## MITOCHONDRIAL DISEASE

Mitochondrial disease comprises a heterogeneous group of disorders, owing to the structural and functional complexity of the mitochondrion itself. Mitochondrial biogenesis and function requires the coordinated effort of over 1100 proteins that are encoded by both the nuclear DNA (nDNA) and the maternally inherited mtDNA. Mitochondrial disorders can be caused by pathogenic mutations in mtDNA or nDNA, resulting in a variety of inheritance patterns, including maternal, autosomal dominant, autosomal recessive and X-linked [39].

Mitochondrial disease symptoms are wide ranging, from mild to severe, early onset to late onset, and can affect one organ or multiple systems. In this regard, mitochondrial disorders do not always show a genotype–phenotype correlation. Two patients may possess the same pathogenic mutation but present differently, or have different mutations that result in the same clinical phenotype. For example, mutations in *MT-ND1* and *MT-ND4* can cause Leber hereditary optic neuropathy (LHON), a disease characterized by bilateral, painless central vision loss in early to late adulthood resulting from the specific degeneration of retinal ganglion cells in the optic nerve [40]. However, these same mutations can also be associated with more severe symptoms, including dystonia and short stature [41–43].

The most common mitochondrial disorders are attributed to dysfunction of the OXPHOS system and occur at an estimated frequency of 1 in 5000 live births. OXPHOS disorders are characterized by deficiencies in OXPHOS complex activity and/or reductions to the steady-state levels of the OXPHOS complexes, with subsequent diminished ATP production. These may be isolated complex disorders or a combination thereof. Defects can be caused by mutations in genes that encode protein subunits of the OXPHOS complexes, proteins required for OXPHOS complex biogenesis, as well as proteins that are vital for the replication, transcription and translation of mtDNA (Table 1) [39].

**Table 1:** Genes with pathogenic mutations resulting in OXPHOS disorders

	Complex subunits	Proteins for import/processing or assembly	mtDNA expression or replication	Nucleotide transport or synthesis	Membrane composition
Complex I	*MT-ND1, MT-ND2, MT-ND3, MT-ND4, MT-ND4L, MT-ND5, MT-ND6, NDUFA1, NDUFA2, NDUFA4, NDUFA8, NDUFA9, NDUFA10, NDUFA11, NDUFA12, NDUFB3, NDUFB8, NDUFB9, NDUFS1, NDUFS2, NDUFS3, NDUFS4, NDUFS6, NDUFS7, NDUFS8, NDUFV1, NDUFV2, NDUFV3*	*ACAD9, FOXRED1, NDUFAF1, NDUFAF2, NDUFAF3, NDUFAF4, NDUFAF5, NDUFAF6, NUBPL*	*AARS2, AGK, C10orf2, C12orf65, DARS2, EARS2, FARS2, GFM1, GFM2, LRPPRC, MPV17, MRPL3, MRPS16, MRPS22, MT01, MTFMT, MTPAP, MTTL1, MTTW, POLG, POLG2, RARS2, RMND1, SARS2, TACO1, TRMU, TSFM, TUFM, YARS2*	*DGUOK, POS1, RRM2B, SLC25A4, SLC25A3, SUCLA2, SUCLG1, TK2, TYMP*	*CABC1, COQ2, COQ6, COQ9, CYCS, DNM1L, MPC1 MFN2, NMT, OPA1, PDSS1, PDSS2, SERAC1, TAZ*
Complex II	*SDHA, SDHB, SDHC, SDHD*	*SDHAF1, SDHAF2*			
Complex III	*CYC1,MT-CYB, UQCRB, UQCRQ, UQCRC2*	*BCS1L, HCCS, LYRM7, TTC19, UQCC2, UQCC3*			
Complex IV	C*OX4I2, COX6B1, COX7B, MT-CO1, MT-CO2, MT-CO3*	*APOPT1, COA5, COA6, COX10, COX14, COX15, COX20, ETHE1, FASTKD2, PET100, SCO1, SCO2, SURF1*			
Complex V	*ATP5E, MT-ATP6, MT-ATP8*	*ATPAF2, TMEM70*			
Multiple complexes		*ABCB7, AFG3L2, BOLA3, DNAJC19, FXN, GFER, GLRX5, HSPD1, ISCU, LYRM4, NFU1, SPG7, TIMM8A*			

### OXPHOS complex I

Isolated CI deficiencies (OMIM #252010) are the most common causes of OXPHOS disorders, accounting for approximately 30% of affected patients. CI diseases comprise a range of clinically heterogeneous conditions. Complex I consists of 44 different structural subunits, one of which is found twice, for a total of 45 subunits. Pathogenic mutations have been identified in 21 CI subunit genes, including all seven of the mtDNA-encoded subunits [44,45] (Table 1).

In recent years, significant advances have also been made in our understanding of the machinery involved in CI biogenesis. This process requires additional proteins, termed ‘assembly factors’, which aid complex I assembly [46]. The first pathogenic human mutation in a CI assembly factor was found in *NDUFAF2* [47]. Since this initial discovery, several other pathogenic mutations have been identified in CI assembly factor genes, including *NDUFAF1* [48], *NDUFAF3* [49], *NDUFAF4* [50], *NDUFAF5* [51], *NDUFAF6* [52], *FOXRED1* [53], *ACAD9* [54] and *NUBPL* [53] (Table 1). The identification of these mutations, and the examination of how they disrupt CI assembly, have greatly aided our understanding of CI biogenesis.

The clinical presentation of CI disorders can range from the neonatal period to adult onset, with symptoms including cardiomyopathy, liver disease and neurological disorders [55–58]. The most common clinical presentations include Leigh Syndrome, a multiple organ disorder with degeneration of the muscular, peripheral and central nervous systems [55,59–61], fatal infantile lactic acidosis and other infancy/early childhood onset neuropathological disorders [44].

In addition, CI defects have been linked to Parkinson's disease (PD), a neurodegenerative condition which results from the progressive loss of dopaminergic neurons in the substantia nigra. Isolated CI respiratory deficiencies are a common feature, specifically in the substantia nigra of PD patients [62,63]. The underlying pathogenic mechanisms are not well understood, although a growing body of research suggests mitochondrial respiratory dysfunction and oxidative stress contribute to disease pathogenesis [62–65]. In some Parkinson's disease patients, mtDNA mutations have also been detected, specifically in the D-loop region and the *MT-ND5* gene [65].

### OXPHOS complex II

Complex II (CII) is composed of four highly conserved nuclear encoded subunits and participates in both OXPHOS and the TCA cycle. In OXPHOS, CII transfers electrons to reduce ubiquinone, whereas in the TCA cycle it metabolizes fumarate to succinate.

Although CII deficiencies (OMIM # 252011) are rare, they still demonstrate the typical clinical heterogeneity associated with mitochondrial diseases. Many CII disorders present in childhood as retinopathies [66] or as encephalopathies [67,68], namely Leigh Syndrome [69], with accompanying cardiomyopathy [70]. Conversely, adult onset CII disorders have also been reported [71]. Mutations in the CII subunit genes *SDHA* [72], *SDHB* [73], *SDHC* [74] and *SDHD* [75] have also been associated with paragangliomas and pheochromocytoma (Table 1).

Pathogenic mutations in CII assembly factors have also been identified, including *SDHAF1* [67,76], which can result in infantile leukoencephalopathy [76], and *SDHAF2* [77] (Table 1). *SDHAF2* mutations may present similarly to mutations in *SDHA* [72], *SDHB* [73], *SDHC* [74] and *SDHD* [75] as paraganglioma and pheochromocytoma [77,78].

### OXPHOS complex III

Complex III (CIII) subunits are largely encoded by the nDNA with only one being mtDNA encoded, cytochrome *b* [79]. Conditions attributed to CIII deficiency (OMIM **#**124000) are uncommon, with wide ranging clinical variability. Clinical presentations can include lactic acidosis, sensorineural loss, liver failure, LHON, developmental delay, cardiomyopathies and encephalopathy [80–83]. In addition, mutations in genes encoding CIII assembly factors have also been identified [84,85]. For example, mutations in *BCS1L*, which encodes the mitochondrial chaperone BCS1, disrupt CIII assembly with increased reactive oxygen species production, resulting in a severe multiple systems condition [84] (Table 1).

### OXPHOS complex IV

Complex IV (CIV) is the terminal enzyme of the respiratory chain and possesses 13 subunits, three of which are encoded by mtDNA. These three mtDNA-encoded subunits form the catalytic site of CIV [60]. Interestingly, mutations to these three subunits are rare. First identified in 1977 [86], CIV deficiencies (OMIM # 220110) can present as myopathies, facial dimorphism and lactic acidosis [86,87]. The majority of known pathogenic mutations are in nDNA genes that encode CIV structural subunits or assembly factors [60], including COX6B1 [88], and PET100 [89] (Table 1).

### OXPHOS complex V

Complex V (CV) is made up of two functional units that span the inner mitochondrial membrane (F_o_) and the mitochondrial matrix (F_1_). Complex V phosphorylates ADP to produce ATP by utilizing the electrochemical gradient produced during CI to CIV electron transfer [90].

Human pathologies arising from isolated CV deficiencies are the rarest of the OXPHOS disorders [91]. Pathogenic mutations to *MTATP6* [92] can result in maternally inherited Leigh syndrome (MILS) [93] and neuropathy, ataxia, retinal pigmentosa (NARP) [90,92]. Additionally, mutations to CV assembly factors, *TMEM70* [94] and *ATPAF2* [95] have been identified and present with cardiomyopathy, hypotonia, intrauterine growth restriction and oligohydramnios [94,95] (Table 1).

## FAO DISEASE

FAO disorders present as a variety of clinical phenotypes and almost always demonstrate an autosomal recessive inheritance pattern. Of note, there is one reported case of a patient with an autosomal dominant *CPT-2* mutation [96] (Table 2). The incidence of FAO disease is believed to be approximately 1:10000, with clinical phenotypes attributed to energy deficiency in tissues that rely heavily on the FAO pathway, such as the heart, liver and skeletal muscle. In infants, where stored glycogen levels are low and metabolic rates are high, FAO is the primary pathway for generating ATP [97,98]. As such, disruption to FAO can result in severe disease during the early stages of life, and can present as intrauterine growth restriction, prematurity, cardiomyopathies, neuropathies, liver failure, rhabdomyolysis, lactacidemia, Reye-like Syndrome (a condition that mimics the metabolic disease Reye Syndrome with hypoglycaemia and hypoketonemia) and neonatal death. In late-onset FAO disease, symptoms can include myopathy and exercise intolerance, cardiac arrhythmias and neuropathy [4]. Of note, women who are heterozygous for an FAO gene mutation in *HADHA* may experience pregnancy induced disease which commonly manifests as preeclampsia and acute liver failure [99,100].

**Table 2:** Proteins involved in mitochondrial fatty acid β-oxidation (FAO)

Protein	Gene	Pathogenic mutation	Clinical presentations
*Carnitine transport cycle and transport*			
Carnitine O-palmitoyltransferase 1A (CPT1A)	*CPT1A*	Yes	Reye-like syndrome, hypoketosis, coma, hyperammonaemia, hypertriglyceridemia, renal tubular acidosis, hypoglycaemia, hepatomegaly, lethargy, hypotonia, hyperemesis, diarrhoea, hyperbilirubinemia, acute fatty liver of pregnancy, hyperemesis
Carnitine O-palmitoyltransferase 1B (CPT1B)	*CPT1B*	No	
Carnitine O-palmitoyltransferase 1C (CPT1C)	*CPT1C*	Yes	Spastic paraplegia
Carnitine O-palmitoyltransferase 2 (CPT2)	*CPT2*	Yes	Hypothermia, lethargy, seizures, hypotonia, cardiomegaly, hyperreflexia, cardiac arrhythmias, lipid accumulation in liver, heart and kidney, polymicrogyria in brain, microcephaly
Carnitine acylcarnitine translocase (CACT)	*SLC25A20*	Yes	Cardiomyopathy, liver dysfunction, apnoea, seizures, tachycardia, hypotension, coma, hypoglycaemia, dicarboxylic aciduria, hypocarnitinemia, < *tex* − *math*/ > hepatomegaly, sudden infant death
Organic cation/carnitine transporter 2	*SLC22A5*	Yes	Systemic carnitine deficiency, hypoketotic hypoglycaemia, skeletal myopathy, cardiomyopathy
*Fatty acid β-oxidation cycle*			
Very-long chain acyl-CoA dehydrogenase (VLCAD)	*ACADVL*	Yes	Rhabdomyolysis, hypoglycaemia, myopathy, myoglobinuria, hepatomegaly, cardiomegaly, cardiac arrest, hypotonia, lipid accumulation in various tissues
Long-chain acyl-CoA dehydrogenase (LCAD)	*ACADL*	No	
Medium-chain acyl-CoA dehydrogenase (MCAD)	*ACADM*	Yes	Sudden Infant Death, hypoglycaemia, lethargy, coma, fatty deposits in liver, Reye-like syndrome, hyperammonaemia, cardiomyopathy
Short chain acyl-CoA dehydrogenase (SCAD)	*ACADS*	Yes	Acidosis, neurological impairment, myopathy, muscle weakness, emesis, failure to thrive, developmental delay, hypertonia, hyperactivity, reduced consciousness
Short/branched chain specific acyl-CoA dehydrogenase, mitochondrial (SBCAD)	*ACADSB*	Yes	2-Methylbutyryl glycinuria
Mitochondrial trifunctional protein (MTP)			
Long chain enoyl-CoA hydratase (LCEH)	*HADHA*	Yes	Cardiomyopathy, Reye-like Syndrome, liver dysfunction, myopathy, rhabdomyolysis, metabolic acidosis, neuropathy, maternal HELLP syndrome, preeclampsia, acute liver failure of pregnancy, developmental delay, myoglobinuria, hypoparathyroidism
Long-chain 3-hydroxyacyl-CoA dehydrogenase (LCHAD)	*HADHA*	Yes	
Long-chain 3-ketoacyl-CoA thiolase (LCKAT)	*HADHB*	Yes	
3-Ketoacyl-CoA thiolase (KAT)	*ACAA2*	No	
Hydroxyacyl-CoA dehydrogenase (HADH)	*HADH*	Yes	Familial hyperinsulinaemic hypoglycaemia
*Others*			
Acyl-CoA dehydrogenase 9 (ACAD9)	*ACAD9*	Yes	Leigh Syndrome, complex I deficiency, cardiomyopathy, muscle weakness, metabolic acidosis
Acyl-CoA dehydrogenase 10 (ACAD10)	*ACAD10*	No	
Acyl-CoA dehydrogenase 11 (ACAD11)	*ACAD11*	No	
Electron transfer flavoprotein (ETF)	*ETFA, ETFB*	Yes	Glutaric aciduria 2A and 2B, multiple Acyl-CoA dehydrogenase deficiency, isolated myopathy
Electron transfer flavoprotein: ubiquinone oxidoreductase (ETF-QO)	*ETFDH*	Yes	Glutaric aciduria 2C, multiple acyl-CoA dehydrogenase deficiency
Enoyl-CoA hydratase, short chain 1 (ECHS1)	*ECHS1*	Yes	Development delay, cardiomyopathy, apnoea, Leigh syndrome
Enoyl-CoA delta isomerase, 1 (ECI1)	*ECI1*	No	
Enoyl-CoA delta isomerase, 2 (ECI2)	*ECI2*	No	
2,4-Dienoyl-CoA reductase (DECR1)	*DECR1*	No	
Delta(3,5)-delta(2,4)-dienoyl-CoA isomerase, mitochondrial	*ECH1*	No	
propionyl-CoA carboxylase (PCC)	*PCCA, PCCB*	Yes	Propionic academia type I and II, episodic vomiting, lethargy, ketosis, neutropenia, thrombocytopenia, hyperglycinuria, hyperglycinaemia, hypogammaglobulinemia, developmental delay, protein intolerance
Methylmalonyl-CoA epimerase (MCEE)	*MCCE*	Yes	Methylmalonic aciduria, retarded motor development, spasticity, dystonia, failure to thrive, gastroesophageal reflux, metabolic acidosis, dehydration, tachypnea, ketonuria, hydrocephalus and macrocephaly
Methylmalonyl-CoA mutase (MCM)	*MUT*	Yes	Methylmalonic aciduria type mut, poor feeding, dehydration, metabolic acidosis, valine intolerance, lethargy, ketoacidosis, multi-organ failure, developmental delay, interstitial nephritis, seizures, basal ganglia infarct

In addition to the primary FAO enzyme deficiency, disruption of FAO can result in excess metabolic intermediates in affected tissues, including the heart, liver, brain and eyes. This is considered to contribute to organ dysfunction due to the toxicity of these intermediates. Excess intermediates are often found in the blood, and can also be expelled in the urine of affected individuals (and as such are used as biomarkers of FAO disease [4,101]).

The first recognized case of human FAO disease was described in 1973 as a CPT2 deficiency in muscle cells [102]. Since then, mutations in at least 19 transport proteins and enzymes involved in FAO have been identified (Table 2). FAO disease presentation is not always persistent and can appear in bouts. These bouts are triggered by stimuli that require fatty acid breakdown, including fasting, endurance exercise, cold exposure and increased dietary fat consumption. Disorders of FAO have also been attributed to sudden infant death syndrome (SIDS) fatalities [103], resulting in the employment of newborn screening for many FAO deficiencies. Since its implementation, many asymptomatic newborn babies have been diagnosed with an inborn error in FAO metabolism, with an increase in MCAD deficiency diagnoses [104]. Since many patients die during their first metabolic stress induced episode, quick diagnosis, allowing for immediate treatment, has been praised for reductions in FAO mortalities in recent years [104]. Still, proper management of many FAO disorders is hindered by the inability to recognize clear biochemical abnormalities in asymptomatic patients.

At present, no cures exist for FAO disease, with treatment strategies focusing primarily on reducing fat intake. Fasting is also avoided, particularly by increasing carbohydrate consumption at night before sleep or during illness [4]. For carnitine transport disorders, carnitine supplementation has been tested, although there is some disagreement as to its effectiveness [105,106]. Supplementation with medium chain fatty acids may also be a way to bypass deficiencies in long chain fatty acid metabolism, such as VLCAD deficiencies, but this is yet to be verified [4].

## DISORDERS OF FAO ENZYMES

MCAD deficiency (OMIM #201450) is the most well studied FAO disease [107], with the majority of symptomatic MCAD deficiencies thought to result from a common point mutation (985A>G) in *ACADM* [108]. Clinical presentations include hypotonia, Reye-like Syndrome, seizures, apnoea, hepatomegaly, fever, vomiting, diarrhoea and coma [109]. In the Caucasian population of several western countries, the number of *ACADM* mutation carriers is estimated to be less than 1:110; specifically, England (1:68), Australia (1:71), Denmark (1:100) and the United States (1:107) [110].

VLCAD deficiency (OMIM #201475) can affect multiple tissues, including heart and muscle, as well as the liver, and as such can result in a severe clinical phenotype. VLCAD deficiency can be classified into three main subgroups based on age of onset, each of which correlates closely with clinical severity and prognosis. The first subgroup, with neonatal presentation, is characterized by cardiomyopathy and is frequently fatal in early life. The second subgroup presents in infancy, with hypoketosis and hypoglycaemia, and frequently mimics Reye-Syndrome [111,112]. The third subgroup has a milder phenotype with adolescent to adult onset, with clinical phenotypes including exercise intolerance and myopathies [111].

Deficiencies in the octameric MTP (OMIM #609015) result in mitochondrial disease which is primarily neuropathological. The build-up of FAO intermediates, which result in tissue toxicity, has been proposed as a primary pathogenic factor in these diseases. MTP deficiencies are generally characterized by reductions in the enzymatic activities of all three of its constituent enzymes. However, isolated deficiencies have been identified in LCHAD that are caused by *HADHA* mutations [113,114]. In addition, women who are heterozygous for mutations in *HADHA* commonly present with acute fatty liver failure and haemolysis, elevated liver enzymes and low platelet counts (HELLP) during pregnancy [113]. Similar to VLCAD deficiency, MTP deficiencies also follow a correlation between age and severity. Age of onset varies substantially and reported cases range from mild to severe, affecting newborns to adults [115].

SCAD deficiency (OMIM #20170) displays phenotypic variability, although neurological impairment appears to be a common theme. This suggests that there may be other factors, such as environmental or epigenetic, which can trigger the disease symptoms, resulting in the wide range of clinical variability associated with SCAD deficiency [116,117]. Similar to other FAO diseases, SCAD disease can be divided into subgroups based on age of presentation, which can either be in infancy/early childhood or in late adulthood. In infants, developmental delay, hypotonia and myopathy are common [118]. In addition, ethylmalonic aciduria is a common feature of SCAD deficiency, and as such is a commonly used biomarker of the disease [116].

Pathogenic mutations in *ACADS* have been identified in patients with SCAD deficiency, whereas others carry two *ACADS* polymorphisms (G625A and C511T) that are considered susceptibility variants [116,117]. Newborn screening of *SCAD* has identified these variants in patients with ethylmalonic aciduria, however, they have also been detected in many asymptomatic individuals. As such, the clinical relevance of SCAD deficiencies has now come into question [119].

*ECHS1* encodes a mitochondrial hydratase which catalyses the second step of FAO. In addition, ECHS1 is also involved in the isoleucine and valine pathways, converting methacrylyl-CoA to (S)-3-hydroxyisobutyryl-CoA and acryloyl-CoA to 3-hydroxypropionyl-CoA [120].

ECHS1 deficiency (OMIM #616277) is phenotypically variable, with relatively early onset, ranging from neonatal presentation to early childhood. Almost all of the patients identified to date exhibit bilateral lesions to the brain consistent with the primary OXPHOS disorder, Leigh Syndrome. In addition, cardiomyopathy, developmental delay and metabolic acidosis are common, with death mostly under the age of 1 year [120–124] (Table 3). Given the involvement of ECHS1 in both the amino acid and FAO pathways, build-up of intermediates from both pathways are a common feature [120–125]. Interestingly, ECHS1 has been demonstrated to be most active in the valine pathway and may be expendable in FAO [122].

**Table 3:** Pathogenic *ECHS1* mutations and their associated clinical and biochemical features Biochemical and clinical characteristics of *ECHS1* patients identified to date. A. B.–at birth, R. C.–respiratory chain, n. d.–not determined, CS, centrum semiovale; Pu, putamen; GP, globus pallidus; NC, nuclear caudatus; BG, basal ganglia; SN, substantia nigra and PV, periventricular; do, days old; mo, months old; yo, years old.

	Patient information				Clinical		Biochemistry		
Author	ID	M/F	Age of onset	Age now	Symptoms	Neuroimaging (MRI and MRS)	ECHS1 protein levels	Respiratory chain activity and metabolic enzyme analysis	BN-PAGE
Haack et al., 2015	#MRB166 c.(161G>A); (394G>A) p.(Arg54His); (Ala132Thr)	F	1 yo	Alive 8 yo	Hearing loss, development delay, increased lactate, hypotonia, ataxia	n. d.	n. d.	n. d.	n. d.
	#346 c.(176A>G); (476A>G) p.(Asn59Ser); (Gln159Arg)	F	A. B.	Died 4 mo	Hearing loss, epilepsy, cardiomyopathy, increased lactate	Brain atrophy and white matter abnormalities	Reduced	Reduced CI	n. d.
	#376 c.(98T<C); (176A>G) p.(Phe33Ser); (Asn59Ser)	F	A. B.	Alive 3 yo	Hearing loss, developmental delay, epilepsy, cardiomyopathy, increased lactate	Symmetrical bilateral abnormalities BG	Reduced	Reduced CIV	n. d.
	#42031 c.(197T<C); (449A>G) p.(Ile66Thr); (Asp150Gly)	M	A. B.	Died 11 mo	Hearing loss, optic atrophy, developmental delay, epilepsy, dystonia, cardiomyopathy, excessive 2-methyl-1,3,dihydroxybutyrate	Symmetrical punctiform hyper-sensitivities in CS	Reduced	R. C. normal, reduced pyruvate	n. d.
	#52236 c.(229G>C); (476A>G) p.(Glu77Gln); (Gln159Arg)	F	11 mo	Alive 31 yo	Hearing loss, optic atrophy, wheelchair bound by 9yo, spastic tetra paresis, developmental delay, epilepsy, dystonia, increased lactate	Signal hyper-sensitivities in NC and Pu	Reduced	R. C. normal	n. d.
	#57277 C(161G>A); (431dup) p.(Arg54His); (Leu145Alafs*6)	F	A. B.	Alive 16 yo	Hearing loss, optic atrophy, communicates through voice computer, developmental delay, dystonia, increased lactate	Increased T2-signal in Pu and GP until 2yo	Reduced	R. C. normal	n. d.
	#68552 c.(476A>G); (476A>G) p.(Gln159Arg); (Gln159Arg)	F	A. B.	Died 2.3 yo	Developmental delay, epilepsy, dystonia, increased lactate	Symmetrical white matter abnormalities	n. d.	Reduced CI	n. d.
	#68761 c.(161G>A); (817A>G) p.(Arg54His); (Lys273Glu)	M	A. B.	Died 7.5 yo	Developmental delay, epilepsy, dystonia	Brain atrophy	n. d.	Decreased ATP production	n. d.
	#73663 c.(673T>C); (673T>C) p.(Cys225Arg); (Cys225Arg)	F	A. B.	Alive 2 yo	Developmental delay, epilepsy, cardiomyopathy, increased lactate, increased 2-methyl-1,3,dihydroxybutyrate	Delayed myelination, white matter lesions	Reduced	R. C. normal	n. d.
	#76656 c.(268G<A); (583G>A) p.(Gly90Arg); (Gly195Ser)	F	2 yo	Alive 5 yo	Hearing loss, developmental delay, dystonia, increased 2-methyl-1,3,dihydroxybutyrate	Signal hyper-sensitivities in Pu, GP, NC and PV white matter	n. d.	R. C. normal	n. d.
Yamada et al., 2015	III-2 c.(176A>G); (413C>T) p.(Asn59Ser); (Ala138Val)	F	10 mo	Alive 7 yo	Developmental delay, dystonia, intellectual disability, increased lactate and *N*-acetyl-*S*-(2-carboxypropyl) cysteine	Bilateral hyper-sensitivities to Pu, GP, NC and SN	Reduced	R. C. normal	n. d.
	III-3 c.(176A>G); (413C>T) p.(Asn59Ser); (Ala138Val)	M	7 mo	Died 5 yo	Developmental delay, dystonia, intellectual disability, increased lactate, *N*-acetyl-*S*-(2-carboxypropyl) cysteine and 2-methyl-1,3, dihydroxybutyrate	Bilateral hypersensitivities to Pu, GP, NC and SN	Reduced	R. C. normal	n. d.
Tetrault et al., 2015	P1 c.(583A>G); (583G>A) p.(Thr180Ala); (Gly195Ser)	F	2.5 mo	Died 10 mo	Failure to thrive, developmental delay, nystagmus, reduced pyruvate dehydrogenase activity	Bilateral T2 hyper intensity of BG	n. d.	Pyruvate dehydrogenase reduced. R. C. normal in fibroblasts	Reductions to Complexes I and III in muscle
	P2 c.(583A>G); (713C>T) p.(Thr180Ala); (Ala238Val)	M	2.9 yo	Alive 18 yo	Failure to thrive, developmental delay, dystonia, nystagmus, hearing loss, truncal ataxia, microcephaly, increased lactate	Bilateral hyper sensitivity of BG	n. d.	R. C. normal in fibroblasts	n. d.
	P3 c.(583A>G); (713C>T) p.(Thr180Ala); (Ala238Val)	M	10 mo	Alive 13 yo	Failure to thrive, developmental delay, optic atrophy, hearing loss, nystagmus, truncal ataxia, increased lactate	T2 hyperintensities of the BG	n. d.	R. C. normal in muscle	n. d.
	P4 c.(583A>G); (476A>G) p.(Thr180Ala); (Gln159Arg)	F	6 mo	Alive 12 yo	Failure to thrive, hypotonia, dystonia, optic atrophy, nystagmus, hearing loss, microcephaly, hyperketosis and encephalopathy	Hypersensitivity of BG	n. d.	R. C. normal in muscle	Reductions to Complex III and IV
Ferdinandusse et al., 2015	Patient 1 c.(817A > G); (817A > G) p.(Lys273Glu); (Lys273Glu)	F	A. B.	Died 1 do	Depressed respiration, increased lactate, hyperammonaemia, cardiomyopathy, hepatomegaly, degeneration of white matter in brain, spongy myelinopathy, Alzheimer's type II metabolic gliosis, muscularization in intralobular arterioles (lungs)	Multiple cystic lesions	n. d.	n. d.	n. d.
	Patient 2 c.(817A > G); (817A > G) p.(Lys273Glu); (Lys273Glu)	F	A. B.	Died 2 do	Apnoea, increased lactate, encephalopathy, increased short medium and long chain acylcarnitine, increased triacylglycerols, hypoxic respiratory failure, increased alanine and proline, liver steatosis	Multiple cystic lesions	Reduced	n. d.	n. d.
	Patient 3 c.(433C > T); (476A > G) p.(Leu145Phe); (Gln159Arg)	F	4 mo	Alive 7 yo	Hypotonia, developmental delay, microcephaly, hearing loss, dysphagia, apnoea, cardiomyopathy, oedema, increased lactate, 2-methyl-2,3, dihydroxybutyrate and cysteine and reduced E3 lipoamide dehydrogenase	Symmetrical atrophy of cerebellum and atrophy of grey matter	Reduced	R. C. normal in muscle	n. d.
	Patient 4 c.(673 T > C); (674G > C) p.(Cys225Arg); (Cys225Ser)	M	A. B.	Alive 3 yo	Bilateral glaucoma, psychomotor, failure to thrive, retardation, lower limb hypotonia, upper limb dystonia, Kussmaul breathing, increased lactate, metabolic acidosis, hyperketosis, increased acylcarnitines, increased 2-methyl-2,3, dihydroxybutyrate, increased cysteine	T2 hyperintensities detected in GP, Pu and bilateral symmetrical lesions in CP. Atrophy of the midbrain nuclei	Reduced	Normal pyruvate dehydrogenase and R. C. complex activity in muscle	n. d.
Sakai et al., 2014	Patient 1 c.(2T>G); (5C>T) p.(Met1Arg); (Ala2Val)	M	2 mo	Alive (assumed)	Hearing loss, developmental delay, hypotonia, nystagmus, spasticity, increased lactate	Bilateral T2 hyper intensity of the Pu	Reduced	Patient cells–reduced CI, III and IV. Immortalized myoblasts, reduced CI, IV and V	No differences
Peters et al., 2014	Patient 1 c.(473C>A); (414+3G>C) p.(Ala158Asp)	F	A. B.	Died 4 mo	Apnoea, cardiomyopathy, increased lactate, increased cysteine	Atrophy of the brain and symmetrical T2 hypersensitivity in Pu. Large lactate peak	Reduced	Reduced pyruvate dehydrogenase in patient fibroblasts. R. C. was normal	n. d.
	Patient 2 c.(473C>A); (414+3G>C) p.(Ala158Asp)	M	A. B.	Died 8 mo	Apnoea, hypotonia, developmental delay, nystagmus, cardiomyopathy, increased cysteine	Reduced bilateral myelination of GP and Pu	Reduced	Reduced pyruvate dehydrogenase	n. d.

Aside from enzymes directly involved in FAO catalysis, pathogenic mutations have also been identified in other FAO genes including *ACAD9, ACADSB, ETFA, ETFB, ETFDH, PCCA, PCCB, MCEE, MUT, SLC25A20, HADH, CPT1A, CPT1C and CPT2* [4] (Table 2).

## FAO–OXPHOS PROTEIN INTERACTIONS

Reduced NAD and FADH_2_ produced during FAO pass their electrons to the OXPHOS complexes. Therefore, these pathways share substrates and are linked biochemically. Interestingly, primary disorders of one of these pathways have been shown to inhibit or disturb the other.

These secondary defects are thought to arise from the build-up of toxic intermediates [54,121,123,126–131]. However, there is growing evidence that *physical* links between FAO and OXPHOS proteins exist, raising the possibility that loss of these interactions may cause the secondary defects observed and therefore contribute to disease pathology [113,128–130,132,133]

The first descriptions of FAO–OXPHOS protein interactions were the identification of the FAO proteins, MTP and thiolase, bound to OXPHOS CI [132]. It was hypothesized that these physical associations were necessary for NADH oxidation/reduction coupling and provided an efficient mechanism of substrate channelling [132].

Other findings also suggest that physical interactions exist between OXPHOS and FAO proteins that are crucial for their combined function and stability [54,133,134]. ETF was found in purified complexes from porcine liver mitochondria containing CIII [135]. In rat liver mitochondria, the fatty acid proteins VLCAD, ETF, TFP, LCHAD and MCAD were shown to co-migrate with monomeric CI and the CI/III_2_/IV_1–3_ OXPHOS supercomplex by blue native polyacrylamide gel electrophoresis (BN-PAGE) and by sucrose density gradients [134]. Conversely, isovaleryl-CoA dehydrogenase (IVD), a dehydrogenase involved in amino acid metabolism and not FAO, did not co-migrate with OXPHOS complexes or supercomplexes [134]. High levels of ACAD activity were detected in purified OXPHOS supercomplexes by the addition of straight chain acyl-CoA substrates for VLCAD, LCAD, MCAD and SCAD. Furthermore, when palmitoyl-CoA was added to the sucrose gradient fractions that contained OXPHOS supercomplexes (in conjunction with ETF, NAD and ATP), palmitoyl-CoA was completely metabolized without the accumulation of any FAO intermediates [134]. These findings suggest that FAO–OXPHOS supercomplexes are present in mitochondria and that they are metabolically active structures which can oxidize fatty acids.

## COMBINED LCHAD AND OXPHOS DEFECTS

Given the direct physical interactions between FAO and OXPHOS proteins, it is possible that FAO protein defects will affect OXPHOS CI and/or OXPHOS supercomplex respiratory activity. Indeed, patients with LCHAD deficiency frequently exhibit secondary OXPHOS CI deficiencies [127,136]. This is associated with enlarged mitochondria and increased mitochondrial proliferation, which may occur as part of a compensatory mechanism [126,136,137]. LCHAD deficient patients usually present with elevated FAO intermediates and excretion of organic acids in the urine [126], with a combination of FAO/CI clinical phenotypes, including severe hypotonia, developmental delay, seizures and hepatic dysfunction.

As LCHAD interacts with OXPHOS CI and the OXPHOS supercomplex directly [134], it is possible that a primary LCHAD deficiency will disrupt the stability and/or function of CI, resulting in secondary CI defect. Another possible mechanism by which LCHAD deficiency may alter OXPHOS CI stability is via the interaction with cardiolipin.

Cardiolipin has been shown to be vital for OXPHOS complex and supercomplex formation and stability [19–21]. As described previously, mutations in *TAZ*, which encodes the mitochondrial cardiolipin acyl-transferase, Tafazzin, result in reduced levels of mature tetralinoleoylcardiolipin in the inner mitochondrial membrane, with the subsequent destabilization of OXPHOS CI/CIII_2_/CIV_1–3_ supercomplex and monomeric CI [19].

LCHAD also exhibits acyl-CoA acyltransferase activity, and can generate mature tetralinoleoylcardiolipin from its precursor, monolysocardiolipin, in the presence of lineolyl-CoA, oleoyl-CoA and palmitoyl-CoA. Interestingly, the overexpression of LCHAD in Barth Syndrome patient lymphoblasts increases mature cardiolipin production, with an associated increase in the steady-state level of OXPHOS complex subunits [133]. Conversely, knockdown of LCHAD in Barth Syndrome patient lymphoblasts results in the accumulation of monolysocardiolipin [133]. Therefore, LCHAD may be essential for OXPHOS CI/CIII_2_/CIV_1–3_ supercomplex and CI monomer stability and assembly via its ability to generate mature cardiolipin [19,133].

## COMBINED ECHS1 DEFICIENCY AND OXPHOS COMPLEX DEFECTS

Of the 23 patients described thus far with reported ECHS1 deficiency, 19 have undergone OXPHOS respiratory analysis, with six patients exhibiting detectable OXPHOS enzyme defects. Notably, these OXPHOS deficiencies are not consistent, and vary from isolated CI or CIV deficiencies to combined CI, CIII, CIV or CI, CIV, CV deficiencies (Table 3).

BN-PAGE analysis has been performed using cells from three different ECHS1 patients, again with varied results. Reduced steady-state levels were observed in CI and CIII [123], in CIII and CIV [123], or not at all [121]. Of note, the patients with reduced OXPHOS complex steady-state levels did not exhibit OXPHOS enzymatic defects [123]. However, patients with detectable OXPHOS enzymatic defects typically have a more severe form of Leigh Syndrome than patients with no OXPHOS deficits (Table 3).

In addition to the OXPHOS defects, pyruvate dehydrogenase activity was reduced in all but one of the ECHS1 deficiencies that was fatal [120–125] (Table 3). Interestingly, the pyruvate dehydrogenase complex has been shown to bind to CI *in vitro.* It has been suggested that this binding may act to couple NADH oxidation/reduction and increase respiratory efficiency [132]. As such, defects in ECHS1 may disrupt pyruvate dehydrogenase activity and/or stability, resulting in a secondary disruption of OXPHOS CI activity/stability. This may explain in part the CI defects observed in some ECHS1 patients. Alternatively, the accumulation of the toxic intermediates methacylyl-CoA and acryloyl-CoA in ECHS1 deficient patients may be responsible for the pathology observed. These molecules can spontaneously react with sulfhydryl groups, potentially disrupting OXPHOS enzyme complex and pyruvate dehydrogenase activities [120].

## FAO PROTEINS AND OXPHOS COMPLEX I ASSEMBLY

OXPHOS CI is the largest of the respiratory chain complexes. It is a multimeric complex that is arranged into an L-shape structure. This L-shaped structure is composed of a hydrophilic arm and a hydrophobic arm and is highly conserved from bacteria to eukaryotes [138–140]. The proper assembly of mature CI involves the coordinated assembly of 45 structural subunits, the majority of which are encoded by the nDNA and must be transported into the mitochondria via membrane bound transport systems [55,141,142]. Furthermore, a group of proteins termed ‘assembly factors’ are required to form the final, mature holocomplex [143].

One FAO protein that has been identified as a *bona fide* CI assembly factor is acyl-CoA dehydrogenase 9 (ACAD9) [54,128]. ACAD9 was initially identified as a homologue of the FAO protein VLCAD, and can compensate in the absence of VLCAD by producing C12 and C14:1 carnitines [128]. However, Nouws et al. [54] showed that ACAD9 also interacts with two CI assembly factors, NDUFAF1 and Ecsit, and that knockdown of ACAD9 results in altered CI biogenesis and isolated CI deficiencies. Complementation with catalytically inactive ACAD9 was able to rescue CI biogenesis and CI activity in two patients with ACAD9 deficiency (although it was less effective than its wild type counterpart in one patient) [54,128], suggesting that ACAD9 activity is not required for complex I assembly [128]. However, studies have shown that mutations in *ACAD9* affect both FAO and OXPHOS activities simultaneously, suggesting that ACAD9 plays two important roles as both a fatty acid dehydrogenase and a complex I assembly factor.

In addition to ACAD9, the FAO proteins 3-hydroxyacyl-CoA dehydrogenase (HADH) and enoyl-CoA delta isomerase 1 (ECI1) are predicted by phylogenetic profiling to be involved in CI biogenesis [144]. A list of genes that have co-evolved with genes encoding CI subunits (complex I phylogenetic profile (COPP) gene list) contains both *HADH* and *ECI1*, suggesting putative roles for their FAO protein products in CI biogenesis. Interestingly, immunoprecipitation experiments have shown that HADH interacts with the CI subunits NDUFV2 and NDUFS2, further suggesting a role for HADH in CI biogenesis [145]. The COPP gene list also contains genes that have been confirmed experimentally as *bona fide* CI assembly factors, including *FOXRED1* [53], *NDUFAF6* [52] and *NDUFAF5* [51,144], substantiating the validity of the COPP list in predicting the involvement of HADH and ECI1 in CI biogenesis.

## PRIMARY OXPHOS DEFICIENCIES ASSOCIATED WITH SECONDARY FAO DEFECTS

Primary OXPHOS CII deficiencies can result in metabolic disorders associated with secondary defects in FAO, presenting with cardiomyopathy, short stature, lactic acidosis, craniofacial dysmorphic features, hypertrichosis and myopathy [129,130]. Deficiencies in CII have been shown to inhibit FAO, resulting in toxic levels of butyrylcarnitine [129]. It has been suggested that this may be due to disruption of the FAD pool; defects in CII activity will result in FADH_2_ remaining reduced, resulting in a lack of oxidized FAD to accept electrons from the fatty acyl-CoA dehydrogenases involved in the first step of FAO. However, as FAD does not frequently shuttle between the ACADs and CII, this mechanism may only partially explain the combined OXPHOS CII and FAO defects observed in some patients [129]. Alternatively, physical interactions between CII and enzymes involved in FAO may exist, however this is yet to be shown experimentally. Whichever the case, treatment of patients with combined OXPHOS CII/FAO defects has proved problematic, as supplementation with essential fatty acids results in metabolic crises [129].

## CONCLUDING REMARKS

Secondary defects in OXPHOS function, due to primary FAO deficiencies, were initially attributed to reduced co-factor sharing and the build-up of toxic fatty acid intermediates, resulting in the inhibition of OXPHOS complex activities [113,121,123,127,136,146]. Although this may be the case in some FAO disorders, the build-up of toxic intermediates does not sufficiently explain the reduced steady-state levels of OXPHOS complexes observed in patients with mutations in FAO genes [54,123].

However, it has now been shown that FAO proteins interact physically with the OXPHOS complexes [134], and that specific FAO protein defects can result in respiratory chain defects [54,121,127,136,146]. Conversely, primary OXPHOS deficiencies can result in secondary FAO disease [129,130], highlighting the importance of the interactions between the FAO and OXPHOS pathways. Given the large range of metabolic diseases caused by OXPHOS and FAO deficiencies, a detailed understanding of the protein interactions between both pathways will be crucial for our comprehension of the pathogenesis involved and for the development of new therapies for the treatment of mitochondrial disease that will target both the FAO and OXPHOS pathways simultaneously.

## Abbreviations

ACAD9: acyl-CoA dehydrogenase 9
BN-PAGE: blue native polyacrylamide gel electrophoresis
CACT: carnitine acylcarnitine translocase
CI: oxidative phosphorylation complex I (NADH: ubiquinone oxidoreductase, EC 1.6.5.3)
CII: oxidative phosphorylation complex II (succinate: ubiquinone oxidoreductase, EC 1.3.5.1)
CIII: oxidative phosphorylation complex III (ubiquinol: ferricytochrome *c* oxidoreductase, EC 1.10.2.2)
CIV: oxidative phosphorylation complex IV (cytochrome *c* oxidase, EC 1.9.3.1)
COPP: complex one phylogenetic profile
CPT1: carnitine O-palmitoyltransferase 1
CPT2: carnitine O-palmitoyltransferase 2
CV: OXPHOS complex V (F_o_F_1_-ATP synthetase, EC; 3.6.3.14)
ECHS1: enoyl-CoA hydratase, short chain 1, mitochondrial
ECI1: enoyl-CoA delta isomerase, 1
ETF: electron transfer flavoprotein
ETFA: electron transfer flavoprotein alpha subunit
ETFB: electron transfer flavoprotein beta subunit
ETF-QO: electron transfer flavoprotein: ubiquinone oxidoreductase
FAD: flavin adenine dinucleotide
FADH_2_: reduced flavin adenine dinucleotide
FAO: fatty acid β-oxidation
H_2_O: water
HADH: hydroxyacyl-CoA dehydrogenase
KAT: 3-ketoacyl-CoA thiolase, mitochondrial
LCAD: long chain acyl-CoA dehydrogenase
LCEH: long chain enoyl-CoA hydratase
LCHAD: long chain 3-hydroxyacyl-CoA dehydrogenase
LHON: Leber hereditary optic neuropathy
LKAT: long chain 3-ketoacyl-CoA thiolase
MCAD: medium chain acyl-CoA dehydrogenase
MCEE: methylmalonyl-CoA epimerase
MCM: methylmalonyl-CoA mutase
MTP: mitochondrial trifunctional protein
NARP: neuropathy, ataxia, retinal pigmentosa
nDNA: nuclear DNA
NUBPL: nucleotide-binding protein-like
O_2_: oxygen
OXPHOS: oxidative phosphorylation
PCC: propionyl-CoA carboxylase
PCCA: propionyl-CoA carboxylase alpha subunit
PCCB: propionyl-CoA carboxylase beta subunit
PD: Parkinson's disease
SBCAD: short/branched chain specific acyl-CoA dehydrogenase, mitochondrial
SCAD: short chain acyl-CoA dehydrogenase
∆*ψ*_m_: mitochondrial membrane potential
TCA: tricarboxylic acid
VLCAD: very long chain acyl-CoA dehydrogenase

## References

[1] Nunnari J., Suomalainen A. (2012). Mitochondria: in sickness and in health. Cell.

[2] Thorburn D.R., Sugiana C., Salemi R., Kirby D.M., Worgan L., Ohtake A., Ryan M.T. (2004). Biochemical and molecular diagnosis of mitochondrial respiratory chain disorders. Biochim. Biophys. Acta.

[3] Smeitink J., van den Heuvel L., DiMauro S. (2001). The genetics and pathology of oxidative phosphorylation. Nat. Rev. Genet..

[4] Kompare M., Rizzo W.B. (2008). Mitochondrial fatty-acid oxidation disorders. Semin. Pediatr. Neurol..

[5] Dudkina N.V., Kouřil R., Peters K., Braun H.-P., Boekema E.J. (2010). Structure and function of mitochondrial supercomplexes. Biochim. Biophys. Acta.

[6] Hackenbrock C.R., Hochli M., Chau R.M. (1976). Calorimetric and freeze fracture analysis of lipid phase transitions and lateral translational motion of intramembrane particles in mitochondrial membranes. Biochim. Biophys. Acta.

[7] Hatefi Y., Rieske J. (1967). The preparation and properties of DPNH—cytochrome c reductase (complex I–III of the respiratory chain). Methods Enzymol.

[8] Ragan C., Heron C. (1978). The interaction between mitochondrial NADH-ubiquinone oxidoreductase and ubiquinol-cytochrome c oxidoreductase. Evidence for stoicheiometric association. Biochem. J..

[9] Yu C.A., Yu L., King T.E. (1974). Soluble cytochrome b-c1 complex and the reconstitution of succinate-cytochrome c reductase. J. Biol. Chem..

[10] Berry E.A., Trumpower B.L. (1985). Isolation of ubiquinol oxidase from Paracoccus denitrificans and resolution into cytochrome bc1 and cytochrome c-aa3 complexes. J. Biol. Chem..

[11] Iwasaki T., Matsuura K., Oshima T. (1995). Resolution of the aerobic respiratory system of the thermoacidophilic archaeon, Sulfolobus sp. strain 7: I. The archaeal terminal oxidase supercomplex is a functional fusion of respiratory complexes III and IV with no c-type cytochromes. J. Biol. Chem..

[12] Sone N., Sekimachi M., Kutoh E. (1987). Identification and properties of a quinol oxidase super-complex composed of a bc1 complex and cytochrome oxidase in the thermophilic bacterium PS3. J. Biol. Chem..

[13] Eubel H., Heinemeyer J., Braun H.-P. (2004). Identification and characterization of respirasomes in potato mitochondria. Plant Physiol.

[14] Eubel H., Jansch L., Braun H.P. (2003). New insights into the respiratory chain of plant mitochondria. Supercomplexes and a unique composition of complex II1. Plant Physiol..

[15] Minauro-Sanmiguel F., Wilkens S., Garcia J.J. (2005). Structure of dimeric mitochondrial ATP synthase: novel F0 bridging features and the structural basis of mitochondrial cristae biogenesis. Proc. Natl. Acad. Sci. U.S.A..

[16] Schagger H., Pfeiffer K. (2000). Supercomplexes in the respiratory chains of yeast and mammalian mitochondria. EMBO J..

[17] Acin-Perez R., Bayona-Bafaluy M.P., Fernandez-Silva P., Moreno-Loshuertos R., Perez-Martos A., Bruno C., Moraes C.T., Enriquez J.A. (2004). Respiratory complex III is required to maintain complex I in mammalian mitochondria. Mol. Cell.

[18] Diaz F., Fukui H., Garcia S., Moraes C.T. (2006). Cytochrome c oxidase is required for the assembly/stability of respiratory complex I in mouse fibroblasts. Mol. Cell Biol..

[19] McKenzie M., Lazarou M., Thorburn D.R., Ryan M.T. (2006). Mitochondrial respiratory chain supercomplexes are destabilized in Barth Syndrome patients. J. Mol. Biol..

[20] Eble K.S., Coleman W.B., Hantgan R.R., Cunningham C.C. (1990). Tightly associated cardiolipin in the bovine heart mitochondrial ATP synthase as analyzed by 31P nuclear magnetic resonance spectroscopy. J. Biol. Chem..

[21] Gomez B., Robinson N.C. (1999). Phospholipase digestion of bound cardiolipin reversibly inactivates bovine cytochrome bc1. Biochemistry.

[22] Barth P.G., Scholte H.R., Berden J.A., Van der Klei-Van Moorsel J.M., Luyt-Houwen I.E., Van ’t Veer-Korthof E.T., Van der Harten J.J., Sobotka-Plojhar M.A. (1983). An X-linked mitochondrial disease affecting cardiac muscle, skeletal muscle and neutrophil leucocytes. J. Neurol. Sci..

[23] Barth P.G., Van den Bogert C., Bolhuis P.A., Scholte H.R., van Gennip A.H., Schutgens R.B.H., Ketel A.G. (1996). X-linked cardioskeletal myopathy and neutropenia (Barth syndrome): Respiratory-chain abnormalities in cultured fibroblasts. J. Inherit. Metab. Dis..

[24] Boumans H., Grivell L.A., Berden J.A. (1998). The respiratory chain in yeast behaves as a single functional unit. J. Biol. Chem..

[25] Lapuente-Brun E., Moreno-Loshuertos R., Acin-Perez R., Latorre-Pellicer A., Colas C., Balsa E., Perales-Clemente E., Quiros P.M., Calvo E., Rodriguez-Hernandez M.A. (2013). Supercomplex assembly determines electron flux in the mitochondrial electron transport chain. Science.

[26] Maranzana E., Barbero G., Falasca A.I., Lenaz G., Genova M.L. (2013). Mitochondrial respiratory supercomplex association limits production of reactive oxygen species from complex I. Antioxid. Redox Signal..

[27] Lenaz G., Baracca A., Barbero G., Bergamini C., Dalmonte M.E., Del Sole M., Faccioli M., Falasca A., Fato R., Genova M.L. (2010). Mitochondrial respiratory chain super-complex I–III in physiology and pathology. Biochim. Biophys. Acta.

[28] Genova M.L., Bianchi C., Lenaz G. (2005). Supercomplex organization of the mitochondrial respiratory chain and the role of the Coenzyme Q pool: pathophysiological implications. BioFactors.

[29] Blaza J.N., Serreli R., Jones A.J.Y., Mohammed K., Hirst J. (2014). Kinetic evidence against partitioning of the ubiquinone pool and the catalytic relevance of respiratory-chain supercomplexes. Proc. Natl. Acad. Sci. U.S.A..

[30] Dudkina N.V., Kudryashev M., Stahlberg H., Boekema E.J. (2011). Interaction of complexes I, III, and IV within the bovine respirasome by single particle cryoelectron tomography. Proc. Natl. Acad. Sci. U.S.A..

[31] Galli C., Marangoni F. (1997). Recent advances in the biology of n-6 fatty acids. Nutrition.

[32] Yamashita A., Sugiura T., Waku K. (1997). Acyltransferases and transacylases involved in fatty acid remodeling of phospholipids and metabolism of bioactive lipids in mammalian cells. J. Biochem..

[33] van der Vusse G.J., Glatz J.F., Stam H.C., Reneman R.S. (1992). Fatty acid homeostasis in the normoxic and ischemic heart. Physiol. Rev..

[34] Jansson E., Kaijser L. (1987). Substrate utilization and enzymes in skeletal muscle of extremely endurance-trained men. J. Appl. Physiol..

[35] Kuhajda F.P., Lane W.J.L.D. (2013). Encyclopedia of Biological Chemistry.

[36] Houten S.M., Herrema H., Te Brinke H., Denis S., Ruiter J.P., van Dijk T.H., Argmann C.A., Ottenhoff R., Muller M., Groen A.K. (2013). Impaired amino acid metabolism contributes to fasting-induced hypoglycemia in fatty acid oxidation defects. Hum. Mol. Genet..

[37] Houten S.M., Violante S., Ventura F.V., Wanders R.J. (2016). The biochemistry and physiology of mitochondrial fatty acid beta-oxidation and its genetic disorders. Annu. Rev. Physiol..

[38] Hirsch D., Stahl A., Lodish H.F. (1998). A family of fatty acid transporters conserved from mycobacterium to man. Proc. Natl. Acad. Sci. U.S.A..

[39] Ohtake A., Murayama K., Mori M., Harashima H., Yamazaki T., Tamaru S., Yamashita Y., Kishita Y., Nakachi Y., Kohda M. (2014). Diagnosis and molecular basis of mitochondrial respiratory chain disorders: exome sequencing for disease gene identification. Biochim. Biophys. Acta.

[40] Man P.Y., Turnbull D.M., Chinnery P.F. (2002). Leber hereditary optic neuropathy. J. Med. Genet..

[41] Taylor R.W., Turnbull D.M. (2005). Mitochondrial DNA mutations in human disease. Nat. Rev. Genet..

[42] Jun A.S., Brown M.D., Wallace D.C. (1994). A mitochondrial DNA mutation at nucleotide pair 14459 of the NADH dehydrogenase subunit 6 gene associated with maternally inherited Leber hereditary optic neuropathy and dystonia. Proc. Natl. Acad. Sci. U.S.A..

[43] Shoffner J.M., Brown M.D., Stugard C., Jun A.S., Pollock S., Haas R.H., Kaufman A., Koontz D., Kim Y., Graham J.R. (1995). Leber's hereditary optic neuropathy plus dystonia is caused by a mitochondrial DNA point mutation. Ann. Neurol..

[44] Fassone E., Rahman S. (2012). Complex I deficiency: clinical features, biochemistry and molecular genetics. J. Med. Genet..

[45] Bugiani M., Invernizzi F., Alberio S., Briem E., Lamantea E., Carrara F., Moroni I., Farina L., Spada M., Donati M.A. (2004). Clinical and molecular findings in children with complex I deficiency. Biochim. Biophys. Acta.

[46] Andrews B., Carroll J., Ding S., Fearnley I.M., Walker J.E. (2013). Assembly factors for the membrane arm of human complex I. Proc. Natl. Acad. Sci..

[47] Ogilvie I., Kennaway N.G., Shoubridge E.A. (2005). A molecular chaperone for mitochondrial complex I assembly is mutated in a progressive encephalopathy. J. Clin. Invest..

[48] Dunning C.J., McKenzie M., Sugiana C., Lazarou M., Silke J., Connelly A., Fletcher J.M., Kirby D.M., Thorburn D.R., Ryan M.T. (2007). Human CIA30 is involved in the early assembly of mitochondrial complex I and mutations in its gene cause disease. EMBO J..

[49] Saada A., Vogel R.O., Hoefs S.J., van den Brand M.A., Wessels H.J., Willems P.H., Venselaar H., Shaag A., Barghuti F., Reish O. (2009). Mutations in NDUFAF3 (C3ORF60), encoding an NDUFAF4 (C6ORF66)-interacting complex I assembly protein, cause fatal neonatal mitochondrial disease. Am. J. Hum. Genet..

[50] Saada A., Edvardson S., Rapoport M., Shaag A., Amry K., Miller C., Lorberboum-Galski H., Elpeleg O. (2008). C6ORF66 is an assembly factor of mitochondrial complex I. Am. J. Hum. Genet..

[51] Sugiana C., Pagliarini D.J., McKenzie M., Kirby D.M., Salemi R., Abu-Amero K.K., Dahl H.H., Hutchison W.M., Vascotto K.A., Smith S.M. (2008). Mutation of C20orf7 disrupts complex I assembly and causes lethal neonatal mitochondrial disease. Am. J. Hum. Genet..

[52] McKenzie M., Tucker E.J., Compton A.G., Lazarou M., George C., Thorburn D.R., Ryan M.T. (2011). Mutations in the gene encoding C8orf38 block complex I assembly by inhibiting production of the mitochondria-encoded subunit ND1. J. Mol. Biol..

[53] Calvo S.E., Tucker E.J., Compton A.G., Kirby D.M., Crawford G., Burtt N.P., Rivas M., Guiducci C., Bruno D.L., Goldberger O.A. (2010). High-throughput, pooled sequencing identifies mutations in NUBPL and FOXRED1 in human complex I deficiency. Nat. Genet..

[54] Nouws J., Nijtmans L., Houten S.M., van den Brand M., Huynen M., Venselaar H., Hoefs S., Gloerich J., Kronick J., Hutchin T. (2010). Acyl-CoA dehydrogenase 9 is required for the biogenesis of oxidative phosphorylation complex I. Cell Metab..

[55] Mimaki M., Wang X., McKenzie M., Thorburn D.R., Ryan M.T. (2012). Understanding mitochondrial complex I assembly in health and disease. Biochim. Biophys. Acta.

[56] Bénit P., Chretien D., Kadhom N., de Lonlay-Debeney P., Cormier-Daire V., Cabral A., Peudenier S., Rustin P., Munnich A., Rötig A. (2001). Large-scale deletion and point mutations of the nuclear NDUFV1 and NDUFS1 genes in mitochondrial complex I deficiency. Am. J. Hum. Genet..

[57] Kevelam S.H., Rodenburg R.J., Wolf N.I., Ferreira P., Lunsing R.J., Nijtmans L.G., Mitchell A., Arroyo H.A., Rating D., Vanderver A. (2013). NUBPL mutations in patients with complex I deficiency and a distinct MRI pattern. Neurology.

[58] McFarland R., Kirby D.M., Fowler K.J., Ohtake A., Ryan M.T., Amor D.J., Fletcher J.M., Dixon J.W., Collins F.A., Turnbull D.M. (2004). De novo mutations in the mitochondrial ND3 gene as a cause of infantile mitochondrial encephalopathy and complex I deficiency. Ann. Neurol..

[59] Komen J.C., Thorburn D.R. (2014). Turn up the power–pharmacological activation of mitochondrial biogenesis in mouse models. Br. J. Pharmacol..

[60] Rotig A., Munnich A. (2003). Genetic features of mitochondrial respiratory chain disorders. J. Am. Soc. Nephrol..

[61] Moggio M., Colombo I., Peverelli L., Villa L., Xhani R., Testolin S., Di Mauro S., Sciacco M. (2014). Mitochondrial disease heterogeneity: a prognostic challenge. Acta Myol..

[62] Schapira A.H.V., Cooper J.M., Dexter D., Clark J.B., Jenner P., Marsden C.D. (1990). Mitochondrial complex I deficiency in Parkinson's disease. J. Neurochem..

[63] Keeney P.M., Xie J., Capaldi R.A., Bennett J.P. (2006). Parkinson's disease brain mitochondrial complex I has oxidatively damaged subunits and is functionally impaired and misassembled. J. Neurosci..

[64] Greenamyre J.T., Sherer T.B., Betarbet R., Panov A.V. (2001). Complex I and Parkinson's disease. IUBMB Life.

[65] Lin M.T., Cantuti-Castelvetri I., Zheng K., Jackson K.E., Tan Y.B., Arzberger T., Lees A.J., Betensky R.A., Beal M.F., Simon D.K. (2012). Somatic mitochondrial DNA mutations in early Parkinson and incidental Lewy body disease. Ann. Neurol..

[66] Rivner M.H., Shamsnia M., Swift T.R., Trefz J., Roesel R.A., Carter A.L., Yanamura W., Hommes F.A. (1989). Kearns-Sayre syndrome and complex II deficiency. Neurology.

[67] Ghezzi D., Goffrini P., Uziel G., Horvath R., Klopstock T., Lochmuller H., D'Adamo P., Gasparini P., Strom T.M., Prokisch H. (2009). SDHAF1, encoding a LYR complex-II specific assembly factor, is mutated in SDH-defective infantile leukoencephalopathy. Nat. Genet..

[68] Bugiani M., Lamantea E., Invernizzi F., Moroni I., Bizzi A., Zeviani M., Uziel G. (2006). Effects of riboflavin in children with complex II deficiency. Brain Dev..

[69] Bourgeron T., Rustin P., Chretien D., Birch-Machin M., Bourgeois M., Viegas-Pequignot E., Munnich A., Rotig A. (1995). Mutation of a nuclear succinate dehydrogenase gene results in mitochondrial respiratory chain deficiency. Nat. Genet..

[70] Alston C., Ceccatelli Berti C., Blakely E., Oláhová M., He L., McMahon C., Olpin S., Hargreaves I., Nolli C., McFarland R. (2015). A recessive homozygous p.Asp92Gly SDHD mutation causes prenatal cardiomyopathy and a severe mitochondrial complex II deficiency. Hum. Genet..

[71] Birch-Machin M.A., Taylor R.W., Cochran B., Ackrell B.A., Turnbull D.M. (2000). Late-onset optic atrophy, ataxia, and myopathy associated with a mutation of a complex II gene. Ann. Neurol..

[72] Burnichon N., Briere J.J., Libe R., Vescovo L., Riviere J., Tissier F., Jouanno E., Jeunemaitre X., Benit P., Tzagoloff A. (2010). SDHA is a tumor suppressor gene causing paraganglioma. Hum. Mol. Genet..

[73] Schimke R.N., Collins D.L., Stolle C.A. (2010). Paraganglioma, neuroblastoma, and a SDHB mutation: Resolution of a 30-year-old mystery. Am. J. Med. Genet. Part A.

[74] Schiavi F., Boedeker C.C., Bausch B., Peczkowska M., Gomez C.F., Strassburg T., Pawlu C., Buchta M., Salzmann M., Hoffmann M.M. (2005). Predictors and prevalence of paraganglioma syndrome associated with mutations of the SDHC gene. JAMA.

[75] Cascon A., Ruiz-Llorente S., Cebrian A., Telleria D., Rivero J.C., Diez J.J., Lopez-Ibarra P.J., Jaunsolo M.A., Benitez J., Robledo M. (2002). Identification of novel SDHD mutations in patients with phaeochromocytoma and/or paraganglioma. Eur. J. Hum. Genet..

[76] Ohlenbusch A., Edvardson S., Skorpen J., Bjornstad A., Saada A., Elpeleg O., Gartner J., Brockmann K. (2012). Leukoencephalopathy with accumulated succinate is indicative of SDHAF1 related complex II deficiency. Orphanet J. Rare Dis..

[77] Bayley J.P., Kunst H.P., Cascon A., Sampietro M.L., Gaal J., Korpershoek E., Hinojar-Gutierrez A., Timmers H.J., Hoefsloot L.H., Hermsen M.A. (2010). SDHAF2 mutations in familial and sporadic paraganglioma and phaeochromocytoma. Lancet Oncol..

[78] Baysal B.E., Ferrell R.E., Willett-Brozick J.E., Lawrence E.C., Myssiorek D., Bosch A., van der Mey A., Taschner P.E., Rubinstein W.S., Myers E.N. (2000). Mutations in SDHD, a mitochondrial complex II gene, in hereditary paraganglioma. Science.

[79] Bénit P., Lebon S., Rustin P. (2009). Respiratory-chain diseases related to complex III deficiency. Biochim. Biophys. Acta.

[80] Brown M.D., Voljavec A.S., Lott M.T., Torroni A., Yang C.C., Wallace D.C. (1992). Mitochondrial DNA complex I and III mutations associated with Leber's hereditary optic neuropathy. Genetics.

[81] de Lonlay P., Valnot I., Barrientos A., Gorbatyuk M., Tzagoloff A., Taanman J.W., Benayoun E., Chretien D., Kadhom N., Lombes A. (2001). A mutant mitochondrial respiratory chain assembly protein causes complex III deficiency in patients with tubulopathy, encephalopathy and liver failure. Nat. Genet..

[82] Nogueira C., Barros J., Sa M.J., Azevedo L., Taipa R., Torraco A., Meschini M.C., Verrigni D., Nesti C., Rizza T. (2013). Novel TTC19 mutation in a family with severe psychiatric manifestations and complex III deficiency. Neurogenetics.

[83] Marin-Garcia J., Hu Y., Ananthakrishnan R., Pierpont M.E., Pierpont G.L., Goldenthal M.J. (1996). A point mutation in the cytb gene of cardiac mtDNA associated with complex III deficiency in ischemic cardiomyopathy. Biochem. Mol. Biol. Int..

[84] Hinson J.T., Fantin V.R., Schönberger J., Breivik N., Siem G., McDonough B., Sharma P., Keogh I., Godinho R., Santos F. (2007). Missense mutations in the BCS1L gene as a cause of the Björnstad syndrome. N. Engl. J. Med..

[85] Ghezzi D., Arzuffi P., Zordan M., Da Re C., Lamperti C., Benna C., D'Adamo P., Diodato D., Costa R., Mariotti C. (2011). Mutations in TTC19 cause mitochondrial complex III deficiency and neurological impairment in humans and flies. Nat. Genet..

[86] Van Biervliet J.P., Bruinvis L., Ketting D., De Bree P.K., Van der Heiden C., Wadman S.K. (1977). Hereditary mitochondrial myopathy with lactic acidemia, a De Toni-Fanconi-Debre syndrome, and a defective respiratory chain in voluntary striated muscles. Pediatr. Res..

[87] van Bon B.W., Oortveld M.A., Nijtmans L.G., Fenckova M., Nijhof B., Besseling J., Vos M., Kramer J.M., de Leeuw N., Castells-Nobau A. (2013). CEP89 is required for mitochondrial metabolism and neuronal function in man and fly. Hum. Mol. Genet..

[88] Massa V., Fernandez-Vizarra E., Alshahwan S., Bakhsh E., Goffrini P., Ferrero I., Mereghetti P., D'Adamo P., Gasparini P., Zeviani M. (2008). Severe infantile encephalomyopathy caused by a mutation in COX6B1, a nucleus-encoded subunit of cytochrome c oxidase. Am. J. Hum. Genet..

[89] Lim S.C., Smith K.R., Stroud D.A., Compton A.G., Tucker E.J., Dasvarma A., Gandolfo L.C., Marum J.E., McKenzie M., Peters H.L. (2014). A founder mutation in PET100 causes isolated complex IV deficiency in Lebanese individuals with Leigh syndrome. Am. J. Hum. Genet..

[90] Jonckheere A.I., Smeitink J.A.M., Rodenburg R.J.T. (2012). Mitochondrial ATP synthase: architecture, function and pathology. J. Inherit. Metab. Dis..

[91] Rodenburg R.J. (2011). Biochemical diagnosis of mitochondrial disorders. J. Inherit. Metab. Dis..

[92] Holt I.J., Harding A.E., Petty R.K., Morgan-Hughes J.A. (1990). A new mitochondrial disease associated with mitochondrial DNA heteroplasmy. Am. J. Hum. Genet..

[93] D'Aurelio M., Vives-Bauza C., Davidson M.M., Manfredi G. (2010). Mitochondrial DNA background modifies the bioenergetics of NARP/MILS ATP6 mutant cells. Hum. Mol. Genet..

[94] Spiegel R., Khayat M., Shalev S.A., Horovitz Y., Mandel H., Hershkovitz E., Barghuti F., Shaag A., Saada A., Korman S.H. (2011). TMEM70 mutations are a common cause of nuclear encoded ATP synthase assembly defect: further delineation of a new syndrome. J. Med. Genet..

[95] De Meirleir L., Seneca S., Lissens W., De Clercq I., Eyskens F., Gerlo E., Smet J., Van Coster R. (2004). Respiratory chain complex V deficiency due to a mutation in the assembly gene ATP12. J. Med. Genet..

[96] Ionasescu V., Hug G., Hoppel C. (1980). Combined partial deficiency of muscle carnitine palmitoyltransferase and carnitine with autosomal dominant inheritance. J. Neurol. Neurosurg. Psychiatry.

[97] Hale D.E., Batshaw M.L., Coates P.M., Frerman F.E., Goodman S.I., Singh I., Stanley C.A. (1985). Long-chain acyl coenzyme A dehydrogenase deficiency: an inherited cause of nonketotic hypoglycemia. Pediatr. Res..

[98] Aoyama T., Uchida Y., Kelley R.I., Marble M., Hofman K., Tonsgard J.H., Rhead W.J., Hashimoto T. (1993). A novel disease with deficiency of mitochondrial very-long-chain acyl-CoA dehydrogenase. Biochem. Biophys. Res. Commun..

[99] Yang Z., Zhao Y., Bennett M.J., Strauss A.W., Ibdah J.A. (2002). Fetal genotypes and pregnancy outcomes in 35 families with mitochondrial trifunctional protein mutations. Am. J. Obstet. Gynecol..

[100] Ibdah J.A., Bennett M.J., Rinaldo P., Zhao Y., Gibson B., Sims H.F., Strauss A.W. (1999). A fetal fatty-acid oxidation disorder as a cause of liver disease in pregnant women. N. Engl. J. Med..

[101] Vockley J., Whiteman D.A.H. (2002). Defects of mitochondrial β-oxidation: a growing group of disorders. Neuromuscul. Disord..

[102] DiMauro S., DiMauro P.M. (1973). Muscle carnitine palmityltransferase deficiency and myoglobinuria. Science.

[103] Scalais E., Bottu J., Wanders R.J.A., Ferdinandusse S., Waterham H.R., De Meirleir L. (2015). Familial very long chain acyl-CoA dehydrogenase deficiency as a cause of neonatal sudden infant death: Improved survival by prompt diagnosis. Am. J. Med. Genet..

[104] Wilcken B., Wiley V., Hammond J., Carpenter K. (2003). Screening newborns for inborn errors of metabolism by tandem mass spectrometry. N. Engl. J. Med..

[105] Stoler J.M., Sabry M.A., Hanley C., Hoppel C.L., Shih V.E. (2004). Successful long-term treatment of hepatic carnitine palmitoyltransferase I deficiency and a novel mutation. J. Inherit. Metab. Dis..

[106] Spiekerkoetter U., Lindner M., Santer R., Grotzke M., Baumgartner M.R., Boehles H., Das A., Haase C., Hennermann J.B., Karall D. (2009). Treatment recommendations in long-chain fatty acid oxidation defects: consensus from a workshop. J. Inherit. Metab. Dis..

[107] Grosse S.D., Khoury M.J., Greene C.L., Crider K.S., Pollitt R.J. (2006). The epidemiology of medium chain acyl-CoA dehydrogenase deficiency: an update. Genet. Med..

[108] Blakemore A.I., Singleton H., Pollitt R.J., Engel P.C., Kolvraa S., Gregersen N., Curtis D. (1991). Frequency of the G985 MCAD mutation in the general population. Lancet.

[109] Andresen B.S., Bross P., Udvari S., Kirk J., Gray G., Kmoch S., Chamoles N., Knudsen I., Winter V., Wilcken B. (1997). The molecular basis of medium-chain acyl-CoA dehydrogenase (MCAD) deficiency in compound heterozygous patients: is there correlation between genotype and phenotype?. Hum. Mol. Genet..

[110] Millington D., Koeberl D. (2003). Metabolic screening in the newborn. Growth Genet. Horm..

[111] Andresen B.S., Olpin S., Poorthuis B.J.H.M., Scholte H.R., Vianey-Saban C., Wanders R., Ijlst L., Morris A., Pourfarzam M., Bartlett K. (1999). Clear correlation of genotype with disease phenotype in very-long-chain Acyl-CoA dehydrogenase deficiency. Am. J. Hum. Genet..

[112] Doi T., Abo W., Tateno M., Hayashi K., Hori T., Nakada T., Fukao T., Takahashi Y., Terada N. (2000). Milder childhood form of very long-chain acyl-CoA dehydrogenase deficiency in a 6-year-old Japanese boy. Eur. J. Pediatr..

[113] Tyni T., Palotie A., Viinikka L., Valanne L., Salo M.K., von Döbeln U., Jackson S., Wanders R., Venizelos N., Pihko H. (1997). Long-chain 3-hydroxyacyl–coenzyme A dehydrogenase deficiency with the G1528C mutation: clinical presentation of thirteen patients. J. Pediatr..

[114] Spiekerkoetter U., Sun B., Khuchua Z., Bennett M.J., Strauss A.W. (2003). Molecular and phenotypic heterogeneity in mitochondrial trifunctional protein deficiency due to beta-subunit mutations. Hum. Mutat..

[115] Spierkerkoetter U., Khuchua Z., Yue Z., Strauss A.W. (2004). The early-onset phenotype of mitochondrial trifunctional protein deficiency: a lethal disorder with multiple tissue involvement. J. Inherit. Metab. Dis..

[116] Corydon M.J., Vockley J., Rinaldo P., Rhead W.J., Kjeldsen M., Winter V., Riggs C., Babovic-Vuksanovic D., Smeitink J., De Jong J. (2001). Role of common gene variations in the molecular pathogenesis of short-chain acyl-CoA dehydrogenase deficiency. Pediatr. Res..

[117] Gregersen N., Winter V.S., Corydon M.J., Corydon T.J., Rinaldo P., Ribes A., Martinez G., Bennett M.J., Vianey-Saban C., Bhala A. (1998). Identification of four new mutations in the short-chain acyl-CoA dehydrogenase (SCAD) gene in two patients: one of the variant alleles, 511C–>T, is present at an unexpectedly high frequency in the general population, as was the case for 625G–>A, together conferring susceptibility to ethylmalonic aciduria. Hum. Mol. Genet..

[118] Gregersen N., Andresen B.S., Corydon M.J., Corydon T.J., Olsen R.K., Bolund L., Bross P. (2001). Mutation analysis in mitochondrial fatty acid oxidation defects: Exemplified by acyl-CoA dehydrogenase deficiencies, with special focus on genotype-phenotype relationship. Hum. Mutat..

[119] Wolfe L., Jethva R., Oglesbee D., Vockley J., Pagon R.A., Adam M.P., Ardinger H.H., Wallace S.E., Amemiya A., Bean L.J.H., Bird T.D., Fong C.T., Mefford H.C., Smith R.J.H. (1993). GeneReviews(R).

[120] Peters H., Buck N., Wanders R., Ruiter J., Waterham H., Koster J., Yaplito-Lee J., Ferdinandusse S., Pitt J. (2014). ECHS1 mutations in Leigh disease: a new inborn error of metabolism affecting valine metabolism. Brain.

[121] Sakai C., Yamaguchi S., Sasaki M., Miyamoto Y., Matsushima Y., Goto Y. (2015). ECHS1 mutations cause combined respiratory chain deficiency resulting in Leigh syndrome. Hum. Mutat..

[122] Ferdinandusse S., Friederich M.W., Burlina A., Ruiter J.P.N., Coughlin C.R., Dishop M.K., Gallagher R.C., Bedoyan J.K., Vaz F.M., Waterham H.R. (2015). Clinical and biochemical characterization of four patients with mutations in ECHS1. Orphanet J. Rare Dis..

[123] Tetreault M., Fahiminiya S., Antonicka H., Mitchell G.A., Geraghty M.T., Lines M., Boycott K.M., Shoubridge E.A., Mitchell J.J., Michaud J.L. (2015). Whole-exome sequencing identifies novel ECHS1 mutations in Leigh syndrome. Hum. Mutat..

[124] Haack T.B., Jackson C.B., Murayama K., Kremer L.S., Schaller A., Kotzaeridou U., de Vries M.C., Schottmann G., Santra S., Büchner B. (2015). Deficiency of ECHS1 causes mitochondrial encephalopathy with cardiac involvement. Ann. Clin. Transl. Neurol..

[125] Yamada K., Aiba K., Kitaura Y., Kondo Y., Nomura N., Nakamura Y., Fukushi D., Murayama K., Shimomura Y., Pitt J. (2015). Clinical, biochemical and metabolic characterisation of a mild form of human short-chain enoyl-CoA hydratase deficiency: significance of increased N-acetyl-S-(2-carboxypropyl)cysteine excretion. J. Med. Genet..

[126] Enns G.M., Bennett M.J., Hoppel C.L., Goodman S.I., Weisiger K., Ohnstad C., Golabi M., Packman S. (2000). Mitochondrial respiratory chain complex I deficiency with clinical and biochemical features of long-chain 3-hydroxyacyl-coenzyme A dehydrogenase deficiency. J. Pediatr..

[127] Tyni T. (1996). Pathology of skeletal muscle and impaired respiratory chain function in long-chain 3-hydroxyacyl-CoA dehydrogenase deficiency with the G1528C mutation. Neuromuscul. Disord..

[128] Nouws J., Te Brinke H., Nijtmans L.G., Houten S.M. (2014). ACAD9, a complex I assembly factor with a moonlighting function in fatty acid oxidation deficiencies. Hum. Mol. Genet..

[129] Gargus J.J., Boyle K., Bocian M., Roe D.S., Vianey-Saban C., Roe C.R. (2003). Respiratory complex II defect in siblings associated with a symptomatic secondary block in fatty acid oxidation. J. Inherit. Metab. Dis..

[130] Arpa J., Campos Y., Gutiérrez-Molina M., Cruz-Martinez A., Arenas J., Caminero A.B., Palomo F., Morales C., Barreiro P. (1994). Benign mitochondrial myopathy with decreased succinate cytochrome C reductase activity. Acta Neurol. Scand..

[131] Vockley J., Rinaldo P., Bennett M.J., Matern D., Vladutiu G.D. (2000). Synergistic heterozygosity: disease resulting from multiple partial defects in one or more metabolic pathways. Mol. Genet. Metab..

[132] Sumegi B., Srere P.A. (1984). Complex I binds several mitochondrial NAD-coupled dehydrogenases. J. Biol. Chem..

[133] Taylor W.A., Mejia E.M., Mitchell R.W., Choy P.C., Sparagna G.C., Hatch G.M. (2012). Human trifunctional protein alpha links cardiolipin remodeling to beta-oxidation. PloS One.

[134] Wang Y., Mohsen A.W., Mihalik S.J., Goetzman E.S., Vockley J. (2010). Evidence for physical association of mitochondrial fatty acid oxidation and oxidative phosphorylation complexes. J. Biol. Chem..

[135] Parker W.D., Filley C.M., Parks J.K. (1990). Cytochrome oxidase deficiency in Alzheimer's disease. Neurology.

[136] Das A.M., Fingerhut R., Wanders R.J., Ullrich K. (2000). Secondary respiratory chain defect in a boy with long-chain 3-hydroxyacyl-CoA dehydrogenase deficiency: possible diagnostic pitfalls. Eur. J. Pediatr..

[137] Tonin A.M., Amaral A.U., Busanello E.N., Grings M., Castilho R.F., Wajner M. (2013). Long-chain 3-hydroxy fatty acids accumulating in long-chain 3-hydroxyacyl-CoA dehydrogenase and mitochondrial trifunctional protein deficiencies uncouple oxidative phosphorylation in heart mitochondria. J. Bioenerg. Biomembr..

[138] Friedrich T., Bottcher B. (2004). The gross structure of the respiratory complex I: a Lego System. Biochim. Biophys. Acta.

[139] Duarte M., Pópulo H., Videira A., Friedrich T., Schulte U. (2002). Disruption of iron-sulphur cluster N2 from NADH: ubiquinone oxidoreductase by site-directed mutagenesis. Biochem. J..

[140] Grigorieff N. (1998). Three-dimensional structure of bovine NADH:ubiquinone oxidoreductase (complex I) at 22 A in ice. J. Mol. Biol..

[141] Lazarou M., Thorburn D.R., Ryan M.T., McKenzie M. (2009). Assembly of mitochondrial complex I and defects in disease. Biochim. Biophys. Acta.

[142] Schiff M., Haberberger B., Xia C., Mohsen A.W., Goetzman E.S., Wang Y., Uppala R., Zhang Y., Karunanidhi A., Prabhu D. (2015). Complex I assembly function and fatty acid oxidation enzyme activity of ACAD9 both contribute to disease severity in ACAD9 deficiency. Hum. Mol. Genet..

[143] McKenzie M., Ryan M.T. (2010). Assembly factors of human mitochondrial complex I and their defects in disease. IUBMB Life.

[144] Pagliarini D.J., Calvo S.E., Chang B., Sheth S.A., Vafai S.B., Ong S.-E., Walford G.A., Sugiana C., Boneh A., Chen W.K. (2008). A mitochondrial protein compendium elucidates complex I disease biology. Cell.

[145] Narayan S.B., Master S.R., Sireci A.N., Bierl C., Stanley P.E., Li C., Stanley C.A., Bennett M.J. (2012). Short-chain 3-hydroxyacyl-coenzyme A dehydrogenase associates with a protein super-complex integrating multiple metabolic pathways. PloS One.

[146] Tyni T., Majander A., Kalimo H., Rapola J., Pihko H. (1996). Pathology of skeletal muscle and impaired respiratory chain function in long-chain 3-hydroxyacyl-CoA dehydrogenase deficiency with the G1528C mutation. Neuromuscul. Disord..

